# E6 Oncoproteins from High-Risk Human Papillomavirus Induce Mitochondrial Metabolism in a Head and Neck Squamous Cell Carcinoma Model

**DOI:** 10.3390/biom9080351

**Published:** 2019-08-08

**Authors:** Alfredo Cruz-Gregorio, Ana Karina Aranda-Rivera, Omar Emiliano Aparicio-Trejo, Iris Coronado-Martínez, José Pedraza-Chaverri, Marcela Lizano

**Affiliations:** 1 Unidad de Investigación Biomédica en Cáncer, Instituto Nacional de Cancerología, México/Instituto de Investigaciones Biomédicas, Universidad Nacional Autónoma de México. San Fernando No. 22, Col. Sección XVI, Tlalpan, Ciudad de México 14080, Mexico; 2 Posgrado en Ciencias Biológicas, Universidad Nacional Autónoma de México, Ciudad Universitaria, Ciudad de México 04510, Mexico; 3 Departamento de Biología, Facultad de Química, Universidad Nacional Autónoma de México, Ciudad Universitaria, Ciudad de México 04510, Mexico; 4 Programa de Maestría y Doctorado en Ciencias Bioquímicas, Facultad de Química, Universidad Nacional Autónoma de México, Ciudad Universitaria, Ciudad de México 04510, Mexico; 5 Departamento de Medicina Genómica y Toxicología Ambiental, Instituto de Investigaciones Biomédicas, Universidad Nacional Autónoma de México, Ciudad Universitaria, Ciudad de México 04510, Mexico

**Keywords:** HNSCC, HPV16 and HPV18 E6 oncoproteins, mitochondrial metabolism, oxidative stress, DNA damage, cellular respiration, mitochondrial decoupling, oxidative phosphorylation

## Abstract

Head and neck squamous cell carcinoma (HNSCC) cells that are positive for human papillomavirus (HPV+) favor mitochondrial metabolism rather than glucose metabolism. However, the involvement of mitochondrial metabolism in HNSCC HPV+ cells is still unknown. The aim of this work was to evaluate the role of E6 oncoproteins from HPV16 and HPV18 in the mitochondrial metabolism in an HNSCC model. We found that E6 from both viral types abates the phosphorylation of protein kinase B-serine 473 (pAkt), which is associated with a shift in mitochondrial metabolism. E6 oncoproteins increased the levels of protein subunits of mitochondrial complexes (I to IV), as well as the ATP synthase and the protein levels of the voltage dependent anion channel (VDAC). Although E6 proteins increased the basal and leak respiration, the ATP-linked respiration was not affected, which resulted in mitochondrial decoupling. This increase in leak respiration was associated to the induction of oxidative stress (OS) in cells expressing E6, as it was observed by the fall in the glutathione/glutathione disulfide (GSH/GSSG) rate and the increase in reactive oxygen species (ROS), carbonylated proteins, and DNA damage. Taken together, our results suggest that E6 oncoproteins from HPV16 and HPV18 are inducers of mitochondrial metabolism.

## 1. Introduction

Head and neck squamous cell carcinoma (HNSCC) has the sixth place of cancer death and the fourth place of cancer prevalence worldwide, being more predominant in males than in females, presenting a 3:1 ratio [1]. HNSCC is associated with alcohol consumption, smoking, and infection with human papillomavirus (HPV), mainly types 16 and 18, which contribute up to 95% of HNSCC HPV-positive (HPV+) cases [2]. HNSCC HPV+ cases have increased in the last few years [2,3]. HNSCC HPV+ differ from HPV negative (HPV−) cases in genomic profiles, etiology, epidemiology, response to treatments, DNA damage response (DDR), and metabolic processes [4,5,6,7,8]. It has been shown that HNSCC HPV− cells prefer glucose metabolism, rather than oxidative phosphorylation (OXPHOS), in comparison with HNSCC HPV+, which favor OXPHOS [9,10,11]. However, why mitochondrial metabolism prevails in HPV+ cells remains unknown.

Otto Warburg hypothesized that some cancer cell types reprogram their metabolism, favoring the metabolism of glucose (Warburg effect) instead of OXPHOS, because the mitochondria of these cells are damaged [12]. However, it has been shown in other tumor cell types that their mitochondria are intact and fully functional [13]. Moreover, mitochondria are essential for inducing and maintaining the tumoral profile, by performing bioenergetic and biosynthetic processes, such as tricarboxylic acid (TCA) cycle, electron transport system (ETS), OXPHOS, fatty acid oxidation (FAO), synthesis of amino acids, lipids, nucleotides, heme and iron sulfur groups, even in cell death regulation via apoptosis [13,14]. Thus, mitochondria undertake critical functions for metabolism, growth, and cell survival, maintaining a key role in cancer [15].

During HPV infection, the HPV genome can be integrated into the genome of host cells, which promotes the overexpression of the E6 and E7 oncoproteins mainly by the break or deletion of the open reading frame of the viral transcriptional regulator E2 [16,17]. The HPV viral oncoproteins E7 and E6 promote the immortalization and the transformation of host cells through the interaction with a myriad cellular key proteins, such as pRb and p53, respectively, which promotes their degradation [18,19,20]. Moreover, it has been demonstrated that E6 augments the production of reactive oxygen species (ROS) and oxidative stress (OS) [21,22], which is associated with cell transformation [23,24]. On the other hand, mitochondria are the main organelles that produce ROS, with ETS being an important ROS source [25]. The increase in ROS production may activate transcriptional factors that involved in the antioxidant response such as the nuclear factor (erythroid-derived 2)-like 2 (Nrf2) and the forkhead box O3 (FoxO3a), increasing antioxidant enzymes, such as catalase, superoxide dismutase (SOD), and thioredoxin (Trx) to regulate ROS levels [26,27]. However, OS appears if the antioxidant defense is overwhelmed, which causes damage in the DNA, in proteins, lipids, and carbohydrates [28,29]. We hypothesized that E6 influences the phenotype of HNSCC HPV+ cells by altering mitochondrial metabolism since E6 oncoproteins from HPV16 and HPV18 generate OS and have a crucial role in the transformation of host cells, which induces OS and DNA damage. Thus, the aim of this work was to evaluate the role of E6 oncoproteins in mitochondrial metabolism in a head and neck cancer cell model. We found that E6 oncoproteins from HPV16 and HPV18 favor the mitochondrial oxygen consumption and the increase in mitochondrial mass, which was associated with a decrease in the phosphorylation of protein kinase B (Akt) in serine 473 (pAkt). Although E6 increased the basal respiration, the ATP-linked respiration (or OXPHOS linked respiration) was not significantly affected. Nevertheless, E6 proteins increased the respiration leak and mitochondrial decoupling, which was associated with the induction of OS and the increase in DNA damage that were observed in the HPV16 E6 expressing cells.

## 2. Materials and Methods

### 2.1. Cell Culture

The FaDu epithelial cell line was acquired from American Type Culture Collection (ATCC) (Rockville, MD, USA) and was maintained in Eagle’s Minimum Essential Medium (EMEM) that was supplemented with 10% of fetal bovine serum (FBS) in a humidified incubator with 5% CO_2_.

### 2.2. Plasmids

The HPV16 and HPV18 E6 open reading frames were cloned into the p3XFLAG-CMV10 vector (Sigma-Aldrich, St. Louis, MO, USA) by standard PCR techniques. p3XFLAG-CMV10 vector harbors an ampicillin resistance cassette, a geneticin resistance marker, and a CMV promoter. This plasmid encodes three adjacent FLAG^®^ epitopes (Asp-Tyr-Lys-Xaa-Xaa-Asp) for its detection while using the ANTI-FLAG M2 antibody.

### 2.3. Stable Transfections

FaDu epithelial cells were stably transfected with the empty vector (p3X), or plasmids expressing E6 from HPV16 or HPV18. For this, the cells were seeded and transfected in 24-well plates (4 × 10^4^ cells per well) with 2.5 μg of each plasmid, while using 2.5 μL of PolyFect Transfection reagent (Qiagen, Hylden, Germany), according to the manufacturer’s instructions. 24 h post-transfection, for stable selection cells were incubated with 600 μM of Geneticin (G-418, Sigma-Aldrich), for a month. After this time, clones were selected for each condition and they were grown to perform corresponding assays. The expression of the E6 protein was verified for each clone through immunoblot.

### 2.4. Chemicals

Antimycin, dihydroethidium (DHE), sodium l-ascorbate, sodium azide (NaN3), 5,5′-dithio-bis (2 nitrobenzoic) acid (DTNB), glutathione (GSH), glutathione disulfide (GSSG), sodium l-ascorbate, 2′,7′-dichlorofluorescin diacetate, glutathione reductase (GR), tetramethyl-*p*-phenylene diamine (TMPD), diphenylene iodonium (DPI), horseradish peroxidase (HRP), *N*-acetyl-l-cysteine (NAC), nitroblue tetrazolium (NBT), nicotinamide adenine dinucleotide phosphate (NADPH), NADP+, nicotinamide adenine dinucleotide (NADH), rotenone, sodium succinate dibasic, rotenone, sodium phosphate dibasic (Na_2_HPO_4_), sodium phosphate monobasic (NaH_2_PO_4_), sodium dodecyl sulfate (SDS), oligomycin, xanthine, *N*,*N*,*N*,*N*-tetramethyl-*p*-phenylenediamine (TMPD) triethanolamine, xanthine oxidase, and 2-vinylpyridine (2-VP) were purchased from Sigma-Aldrich. Ethanol, hydrochloric acid, ethylacetate, and trichloroacetic acid, hydrogen peroxide (H_2_O_2_), 2,4-dinitrofenilhidrazina (DNPH), and ethylenediaminetetraacetic acid disodium salt dihydrate (EDTA) were obtained from JT Baker (Xalostoc, Edo. Mexico, Mexico). Acrilamide/Bis was purchased from Bio-Rad (Hercules, California, USA). The protease inhibitor cocktail was purchased from Roche Applied Science (Mannheim, Germany). Eagle’s Minimum Essential Medium (EMEM) and fetal bovine serum (FBS) were purchased from ATCC. MitoSOX™ Red was purchased from Thermo Fisher Scientific (Waltham, MA, USA). 2-Morpholin-4-yl-8-phenylchromen-4-one (LY 294002) was purchased from Cell Signaling (Danvers, MA, USA).

### 2.5. Cell Viability

The viability of FaDu cells stably transfected with the different plasmids was evaluated by their mitochondrial activity, using the 3-(4,5-dimethylthiazol-2-yl)-5-(3-carboxymethoxyphenyl)-2-(4-sulfophenyl)-2H-tetrazolium (MTS) assay (Promega, Madison, WI, USA), according to the manufacturer’s instructions. MTS is reduced to formazan salt by the activity of mitochondrial dehydrogenases. The formazan salt was quantified by measuring its absorbance at 490 nm and the absorbance is directly proportional to the viable cells. Cell viability was expressed as a percentage of MTS reduction to formazan salt.

### 2.6. Cell Respirometry.

The oxygen consumption experiments in intact cells was performed while using a high resolution respirometry equipment O2k meter (Oroboros Instruments, Innsbruck, Austria), as previously described [30]. Briefly, measures in stably transfected cells were made using 2 mL of culture medium with 10% FBS. Each experiment was initiated by the addition of the cells; the respiratory parameters were defined as: 1. Basal respiration, corresponding to the oxygen consumption in presence of cells. 2. Leak of the respiration, corresponding to cellular oxygen consumption in presence of 5 μM oligomycin. 3. Respiratory control index (RCI) corresponding to the ratio basal/leak. 4. ATP-linked respiration or OXPHOS linked respiration was calculated by the formula: Basal/Routine-Leak. All of the parameters were corrected by subtracting the non-mitochondrial respiration, which was obtained by the addition of 2 μM rotenone plus 11.25 μM diphenyleneiodonium chloride plus 5 μM antimycin A, and normalized by the number of cells.

### 2.7. Complex I (CI) Linked Respiration and Complex IV (CIV) Activity

CI linked respiration was measured, as previously described [31]. Briefly, we determined the CI linked respiration resting the basal respiration minus the basal respiration inhibited with 2 μM rotenone. The activity of CIV was evaluated in whole cells in culture medium that was supplemented with 2 µM rotenone plus 5 µM antimycin A, the CIV was stimulated by the addition of 0.5 mM TMPD plus 2 mM sodium l-ascorbate and it was corrected by oxygen consumption in the presence of the appropriate inhibitor (100 mM NaN3). The number of cells normalized the results.

### 2.8. Western Blot Analysis

The protein extracts were obtained by adding lysis buffer (Tris 50 mM, NaCl 150 mM, NP-40 1%) to the cells, which were scraped and lysed by pipetting and boiling. Equal amounts of protein were loaded and separated in SDS-PAGE; subsequently, the proteins were transferred onto a nitrocellulose membrane and then incubated with the proper primary antibody. The membranes were blocked while using 5% milk in tris-buffer saline with tween 20 (TBS/T) buffer. Primary antibodies were prepared in TBS/T buffer in the following concentrations: anti-glyceraldehyde 3 phosphate dehydrogenase (GAPDH) 1:1000 (SC-32233, Santa Cruz Biotechnology Inc., Dallas, TX, USA); anti-actinin 1:1000 (SC-17829, Santa Cruz Biotechnologies); anti-SOD2 1:1000 (#13141, Cell Signaling); anti-SOD1 1:1000 (#4266, Cell Signaling); anti-Akt 1:2000 (#2920, Cell Signaling); anti-phospho-Nrf2 Ser40 (pNrf2) 1:500 (ab76026, Abcam, Cambridge, UK); anti-phopho-Akt (Ser473) 1:2000 (#4060, Cell Signaling); anti-FoxO3a 1:1000 (#99199, Cell Signaling); Glutamate-cysteine ligase modifier subunit (GCLM) 1:500 (ab55436, Abcam); anti-catalase 1:500 (#12980, Cell Signaling); anti-gamma H2A histone family member X (γH2AX) 1:1000 (clone JBW301, Merck Millipore Upstade, Burlington, MA, USA); anti-FLAG M2 1:2000 (F1804, Sigma-Aldrich); anti-voltage dependent anion channel (VDAC) 1:1000 (sc-390996, Santa Cruz Biotechnologies); and, anti-total OXPHOS cocktail 1:1000 (ab110413, Abcam), which targets the following proteins: subunit of Complex I (NDUFB8-20 kD), subunit of Complex II (SDHB-30kD), subunit Complex III-Core protein 2 (UQCRC2-48 kD), subunit Complex IV subunit I (MTCO1-40 kD), and subunit Complex V alpha (ATP5A-55 kD). The membranes were washed three times with PBS/T and then incubated with horseradish peroxidase (HRP) coupled to secondary anti-mouse or rabbit antibodies (Santa Cruz Biotechnologies). Membranes were finally developed using the Chemo Luminiscent Reagent (Merck Millipore, Billerica, MA, USA), accordingly to the manufacturer’s instructions. Densitometric analysis was performed while using the ImageJ program ver.1.48h3, National Institutes of Health (NIH).

### 2.9. ROS Quantification

ROS production was measured while using the fluorescent probe, MitoSOX™ Red, dihydroethidium (DHE) and 2′,7′-Dichlorofluorescin diacetate (H_2_DCFDA), as previously described by Pedraza-Chaverri et al. [32]. MitoSOX™ Red is a highly selective fluorogenic dye for mitochondrial superoxide. DHE is oxidized to 2-hydroxyethidium (2-OH-Et) and ethidium (Et) in the cytosol by superoxide anion. Both of the compounds are then retained in the nucleus, which is then stained, due to their DNA binding capacity. H_2_DCFDA is a cell-permeable non-fluorescent probe that is de-esterified intracellularly and turns to highly fluorescent dichlorofluorescein (DCF) upon oxidation by ROS. Subsequently, ROS was identified as a red and green fluorescent signal at the microscope and then quantified by fluorometry. Stably transfected cells were seeded and after forty-eight 5 μM MitoSOX ™ Red, 15 μM DHE and 15 μM H_2_DCFDA were independently added to culture media without phenol red, incubating for 30 min. at 37 °C. The quantitative data of ROS and cell images were collected through the Cytation^TM^ 5 Cell Imaging Multi-Mode Reader from Biotek (Winoosky, VT, USA), which combines digital wide field microscopy with a conventional multi-mode microplate, which provides high sensitivity in ROS quantification. Thus, cellular ROS production was visualized and measured at 510 nm excitation and at 580 nm emission for MitoSOX ™ Red; at 510 nm excitation and 590 nm emission for DHE and 480 nm excitation and at 520 nm emission for DCF. ROS were visualized and then quantified in three independent experiments, while using Gen5™ 3.0 software (https://www.biotek.com/products/software-robotics-software/gen5-microplate-reader-and-imager-software/, Biotek) for data acquisition and analysis.

### 2.10. Protein Carbonyls

Protein carbonyls were evaluated, as previously described by Levine et al. [33]. Briefly, the cell extracts were incubated with 10 mM 2,4-dinitrophenylhydrazine for 1 h, and then proteins were precipitated with 20% trichloroacetic acid. Proteins were washing fourth times with an ethanol-ethyl acetate mixture (1:1 *v*/*v*), solubilized in 6 M guanidine hydrochloride, and the absorbance was measured at 370 nm.

### 2.11. GSH and GSSG Quantification

Total glutathione [glutathione (GSH) + glutathione disulfide (GSSG)] was measured by the enzymatic recycling method that was described by Rahman et al. [34], in which GSH is oxidized by 5,5′-dithiobis-2-nitrobenzoic acid (DTNB) to 5-thio-2-nitrobenzoic acid (TNB, detectable at λ = 412 nm) and TNB glutathione adducts (GS-TNB). Both GS-TNB and GSSG are reduced by glutathione reductase (GR) in the presence of NADPH, to GSH, which in turn is oxidized by DTNB to TNB. Thus, the amount of total glutathione calculated represents the sum of GSH and GSSG. Subsequently, GSSG was evaluated by the enzymatic recycling method that was mentioned above, where samples were previously treated with 2-vinylpyridine (2-VP). 2-VP, that can covalently associate with GSH, remove all reduced glutathione, leaving the oxidized form of glutathione as the only measurable substrate of the assay. Finally, GSH was calculated by subtracting GSSG from the total glutathione (GSH +GSSG). Briefly, the cell extract of each transfection was diluted with 200 μL of potassium phosphate EDTA (KPE) buffer (0.1 M potassium phosphate, 5 mM disodium EDTA, pH 7.5). Afterwards, two separate samples of 20 μL each and treated with 2-VP, were used to measure either total glutathione or GSSG, mixed with DTNB (2.5 mM) and GR (250 U/mL). Finally, β-NADPH was added and the absorbance at λ = 412 nm was measured at intervals of 60 secs, for 2 min. The rate of change in absorbance for each experiment was compared with the GSH or GSSG standards.

### 2.12. Antioxidant Enzyme Activity Assays

The antioxidant enzyme activities were evaluated, as previously described by Cruz-Gregorio et al. [21]. Briefly, SOD activity was spectrophotometrically measured at 560 nm based on nitro blue tetrazolium (NBT) reduction to formazan. The level of protein that inhibits NBT reduction to 50% was defined as one unit of superoxide dismutase (SOD) activity, including the activity of SOD1 and SOD2. Catalase activity was assayed at 240 nm by a method that is based on the decomposition of H_2_O_2_ by catalase contained in the samples. Units of catalase enzymatic activity are expressed as K/mg protein, as previously described by Aebi [35].

### 2.13. Statistical Analysis

All of the experiments were done in triplicate and the data were analyzed as the mean ± SD. ANOVA and the Tukey’s test were used to determine the statistical significance of the experimental condition versus the control.

## 3. Results

### 3.1. E6 Oncoproteins Reduce pAkt

**Figure 1:**
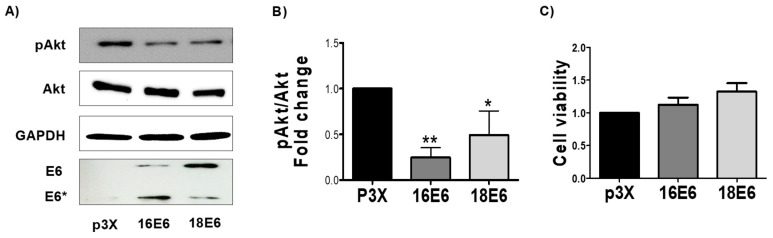
16E6 and 18E6 oncoproteins decrease pAkt without changing cell viability in FaDu cells. FaDu cells were transfected with p3X, HPV16 E6- or HPV18 E6-expressing plasmids and were seeded in a 96 well plate. (**A**) Representative immunoblot; and (**B**) quantitative densitometry showing Flag-tagged E6 and E6* (E6 spliced product), protein kinase B (Akt) phosphorylated in serine 473 (pAkt) and total Akt. Glyceraldehyde 3-phosphate dehydrogenase (GAPDH) was used as a loading control. Human papillomavirus (HPV) E6 oncoproteins reduce pAkt. (**C**) Cell viability was quantified after 48 h by 3-(4,5-dimethylthiazol-2-yl)-5-(3-carboxymethoxyphenyl)-2-(4-sulfophenyl)-2H-tetrazolium (MTS) reduction and expressed as fold change in relation to the control. Data are expressed as the mean ± SD. Tukey’s test * *p* < 0.05 and ** *p* < 0.005 vs. p3X control, n = 3.

Fadu cells were stably transfected with E6 oncoprotein of human papillomavirus 16 or 18 (16E6 or 18E6). Similar E6 protein levels were observed in different clones, as demonstrated by immunoblot through the expression of E6 and E6* (E6 spliced product) from 16E6 and 18E6 in FaDu-transfected cells (Figure 1A). We analyzed the effect of E6 on pAkt protein levels by immunoblot since protein kinase B phosphorylated in serine 253 (pAkt) is a significant driver of glucose metabolism [36,37], whose inhibition increases the activation of mitochondrial metabolism [38]. We found that the pAkt levels were significantly diminished in 1.7 and 1.5-fold in HPV16 and HPV18 E6-transfected cells, respectively (Figure 1A,B), concluding that E6 oncoproteins decreases Akt phosphorylation. To test whether E6 oncoproteins impact FaDu cell viability, we measured cell viability through MTS assay, which measures metabolic activity by tetrazolium reagent reduction to formazan. We found that E6 oncoproteins did not affect FaDu cell viability with respect to the control (Figure 1C).

### 3.2. E6 Oncoproteins Increase Mitochondrial Proteins

**Figure 2:**
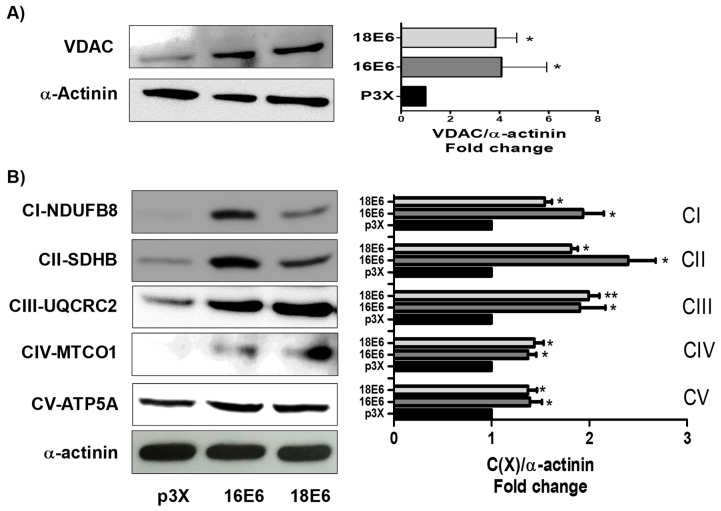
16E6 and 18E6 oncoproteins increase mitochondrial metabolism in FaDu cells. Human papillomavirus (HPV) E6 oncoproteins increase the protein levels of the subunits of mitochondrial complexes I to IV: Complex I subunit CI-NDUFB8, Complex II subunit CII-SDHB, Complex III-Core protein 2 (CIII-UQCRC2), Complex IV subunit I (CIV-MTCO1), and ATP synthase alpha subunit (CIV-ATP5A), as well as the voltage dependence anion channel (VDAC). (**A**) Representative immunoblot and densitometric analysis of VDAC; and (**B**) Representative immunoblot and densitometric analysis of mitochondrial complexes subunits I-IV and ATP synthase subunit in FaDu transfected cells. α-actinin was used as a loading control. Data are expressed as the mean ± SD. Tukey’s test * *p* < 0.05 and ** *p* < 0.005 vs. p3X control, n = 3.

We proceeded to evaluate the relation of the reduction in pAkt levels with mitochondrial proteins. Therefore, voltage dependence anion channel (VDAC) levels were evaluated by immunoblot, as a marker of mitochondrial mass. We found that 16E6 and 18E6 increased VDAC by 4 and 3.8-fold, respectively (Figure 2A), which suggested an increase in mitochondrial mass due to the reduction of pAkt. In order to corroborate such effect, the LY 294,002 a pAkt inhibitor, was tested. We found that a concentration of 25 μM LY 294,002 increases the VDAC protein in FaDu cells that were transfected with the control p3X (Figure S1). Therefore, the effect of E6 proteins in the overexpression of VDAC can be attributed to the decrease in pAkt.

Several models of mitochondrial dysfunction have reported a reduction in protein levels of mitochondrial complexes I to V, as well as in levels of their mRNAs, which have been related to a reduction in mitochondrial biogenesis and bioenergetics in such models [39,40,41,42,43,44]. Therefore, we measured, through immunoblot, the effect of E6 on the subunits of mitochondrial complexes I to V while using an OXPHOS cocktail. We found that 16E6 and 18E6 increased, respectively, the subunit CI-NDUFB8 of complex I in 1.9 and 1.5-fold; the subunit CII-SDHB of complex II in 2.4 and 2.8-fold; the subunit CIII-UQCRC2 of complex III in 1.9 and 1.9-fold; the subunit CIV-MTCO1 of complex IV in 1.4 and 1.5-fold; and, the subunit of CV-ATP5A complex V in 1.4 and 1.4-fold, in relation to the cells that were transfected with the control vector (Figure 2B). Based on all of these results, we conclude that E6 from HPV16 and HPV18 increase the mitochondria mass, especially the proteins of the electron transfer system (ETS), which is related to a reduction in pAkt levels.

### 3.3. E6 Oncoproteins Promote Mitochondrial Electron Transport System Activity

**Figure 3:**
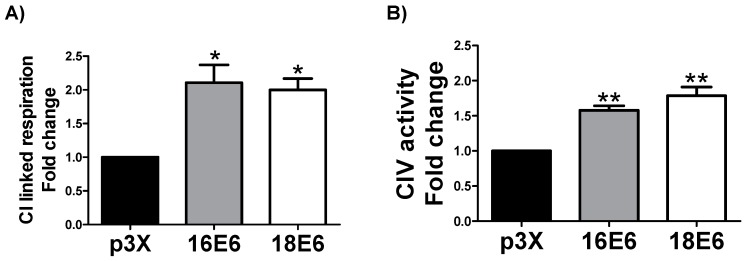
16E6 and 18E6 oncoproteins promote the increase in mitochondrial electron transport system (ETS) activity in FaDu cells. (**A**) Complex I (CI) linked respiration; (**B**) Complex IV (CIV) activity. Data are expressed as the mean ± SD. Tukey’s test * *p* < 0.05 and ** *p* < 0.005 vs. p3X control, n = 3.

The increase in the subunits of mitochondrial complexes suggests changes in mitochondrial bioenergetics, which could be especially related to an increase in the activity of the whole ETS. Accordingly, we evaluated the CI linked respiration and the CIV activity. We found that 16E6 and 18E6 increased CI linked respiration in 2.1 and 2-fold (Figure 3A) and CIV activity in 1.8 and 1.5-fold (Figure 3B), respectively. These results, together with the increase in mitochondrial mass, imply an augment in the activity of whole ETS that is caused by E6 oncoproteins.

### 3.4. E6 Proteins Increase Cellular Respiration Rates, Inducing Mitochondrial Decoupling

**Figure 4:**
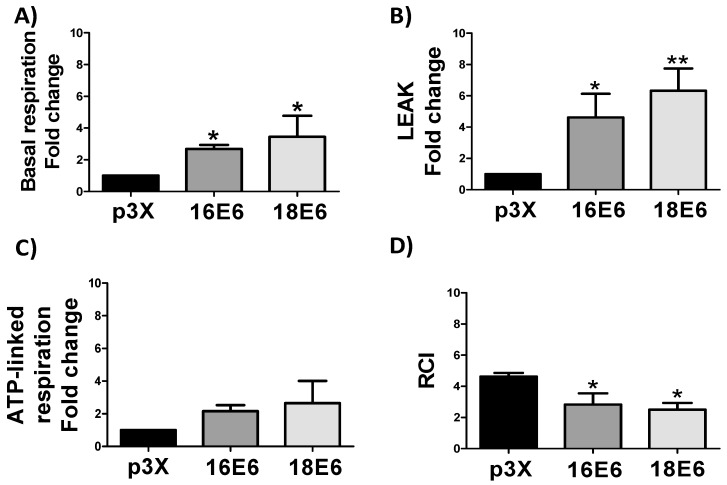
E6 oncoproteins from HPV16 and HPV18 promote cellular respiration and mitochondrial decoupling in FaDu cells. Mitochondrial respiratory parameters: (**A**) basal respiration, (**B**) leak, (**C**) ATP-linked respiration and (**D**) respiratory control index (RCI). Data are expressed as the mean ± SD. Tukey’s test * *p* < 0.05 and ** *p* < 0.005 vs. p3X control, n = 3.

The respiratory parameters were evaluated in whole cells, including ATP-linked respiration, basal and leak respirations, and the respiratory control index (RCI) to determine whether the increase in mitochondrial proteins by E6 is related to mitochondrial bioenergetic. Cell respiration is regulated according to physiological activity, at intracellular adenosine diphosphate (ADP) levels, which is named “basal respiration”. When incubated in the corresponding culture medium, cells maintain a basal level of oxygen consumption activity [45,46]. We found in 16E6 and 18E6 expressing cells an increase of 2.7 and 3.4-fold in basal respiration and 4.6 and 6.3-fold in leak respiration, respectively (Figure 4A,B), which is congruent with the increase in ETS activity and protein levels (Figure 2 and Figure 3). However, the observed increase in leak respiration indicates that only non-phosphorylating respiration is increased, which suggests that the increase in the oxygen consumption that was observed in basal respiration is generated to compensate the electron and proton leak, since ATP-linked respiration did not show significant changes (Figure 4C). Furthermore, RCI parameter displayed a lessening of 1.6 and 1.8-fold, respectively, reaffirming the mitochondrial decoupling (Figure 4D), which suggests that oxygen is not efficiently used by the mitochondria, which could trigger the ROS production.

### 3.5. E6 Promotes Mitochondrial ROS Production

**Figure 5:**
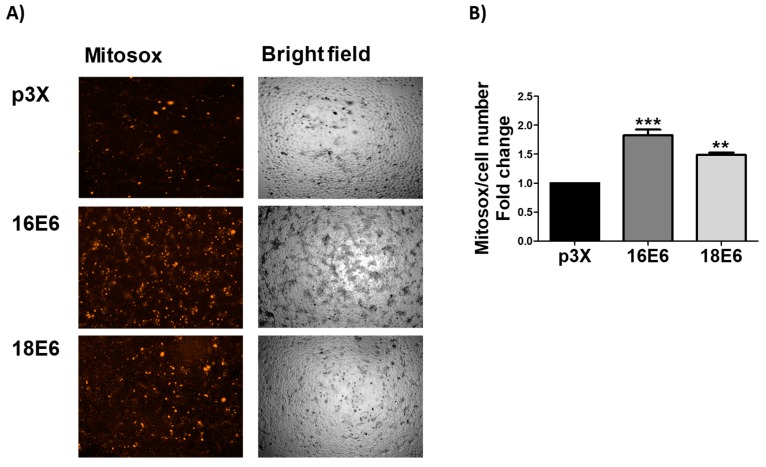
E6 oncoproteins from HPV16 and HPV18 increase reactive oxygen species (ROS) in mitochondria of FaDu cells. (**A**) Representative images and (**B**) quantitative data of ROS generated by E6 proteins from HPV types 16 and 18. Quantitative data were obtained from cells expressing E6 from each viral type, comparing with p3X-transfected cells. The mean intensity of MitoSOX fluorescence was measured using Gen5^TM^ 3.0 software for image acquisition and quantification. The fluorescence intensity is expressed as the mean ± SD. Tukey’s test ** *p* < 0.005 and *** *p* < 0.0005 vs. control (p3X), n = 3.

The respiratory parameter showed that E6 triggered a significant increase in the basal respiration rate. Nevertheless, the increase in respiration leak and the decrease in RCI (Figure 4D) suggest that oxygen is not efficiently used for OXPHOS, which could trigger the increase in mitochondrial ROS production. We use MitoSOX Red assay in order to assess ROS production in the mitochondria. We found an increase in the ROS levels evaluated by Mitosox of 1.8- and 1.5-fold, in the presence of E6 from HPV16 and HPV18 E6, respectively (Figure 5A,B). MitoSOX is meanly oxidized by O_2_^.−^ and exhibits red fluorescence in mitochondria. From this result, we conclude that the induced mitochondrial bioenergetic alterations by E6 oncoproteins (Figure 4) is linked to the increase in ROS production in the mitochondria.

### 3.6. E6 Promotes the Increase in ROS Levels in Whole Cells

**Figure 6:**
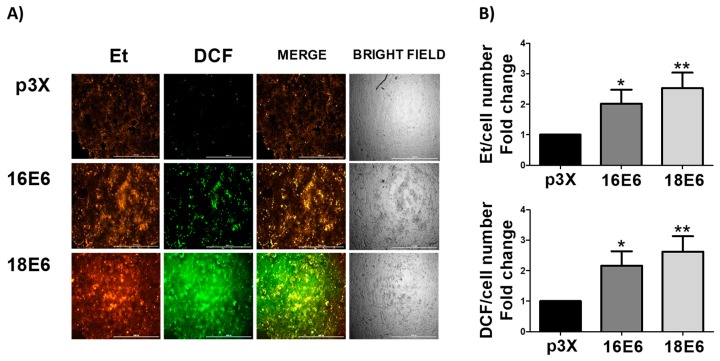
E6 oncoproteins from HPV16 and HPV18 increase reactive oxygen species (ROS) in FaDu cells. (**A**) Representative images and (**B**) quantitative data of ROS generated by E6 proteins from HPV types 16 and 18. Quantitative data were obtained from cells expressing E6 from each viral type, comparing with p3X-transfected cells as control. The mean intensity of ethidium (Et) and dichlorofluorescein (DCF) fluorescence were measured using Gen5^TM^ 3.0 software for image acquisition and quantification. The fluorescence intensity is expressed as the mean ± SD. Tukey’s test * *p* < 0.05, ** *p* < 0.005 and *** *p* < 0.0005 vs. control (p3X), n = 3.

Mitochondrial ROS are released to the cytosol [47,48]. Accordingly, we measured ROS levels in the whole cells. We found an increase in ROS levels of 2- and 2.5-fold, as evaluated by DHE, which is oxidized by O_2_^.−^ to ethidium (Et), in the presence of 16E6 and 18E6, respectively. We measured another ROS by H_2_DCFDA, taking a bigger picture of the ROS production induced by E6, since DHE assay mainly measures O_2_^.−^. The evaluation by DCF showed an increase of 2.2- and 2.7-fold in cells expressing 16E6 and 18E6, respectively (Figure 6A,B).

### 3.7. E6 oncoproteins from HPV16 and HPV18 Induce Oxidative Stress through the Increase in Protein Carbonyls and GSSG and the Decrease in GSH and GSH/GSSG Ratio

**Figure 7:**
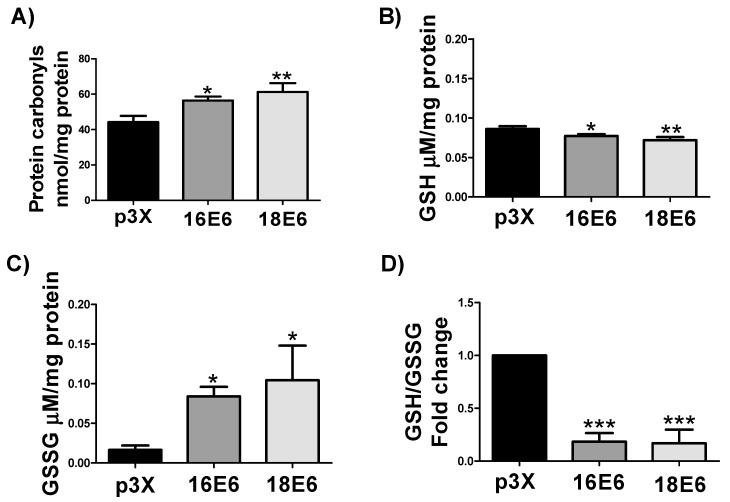
E6 oncoproteins from HPV16 and HPV18 promote oxidative stress in FaDu cells. (**A**) protein carbonyls; (**B**) GSH levels; (**C**) GSSG; and, (**D**) GSH/GSSG ratio, in comparison with the control vector. Protein carbonyls, GSH, and GSSG levels are expressed as the mean ± SD. Tukey’s test * *p* < 0.05, ** *p* < 0.005 and *** *p* < 0.0005.

We decided to measure proteins carbonyls and we found that this OS marker increased with E6 from both HPV types in 1.3- and 1.4-fold, respectively, since ROS can react with proteins to produce protein carbonyls (Figure 7A). This result suggests that E6 oncoprotein induce OS and this was associated with oxidative damage to proteins. Moreover, we evaluated GSH levels as a main marker of OS and we found that both E6 proteins significantly decreased GSH levels (Figure 7B), while the GSSG levels augmented (Figure 7C), resulting in a decrement in GSH/GSSG ratio of 1.8-fold for both proteins (Figure 7D). We concluded that E6 oncoproteins induce OS in whole cells from this result.

### 3.8. E6 from HPV16 and HPV18 Increase pNrf2 and FoxO3a Levels

**Figure 8:**
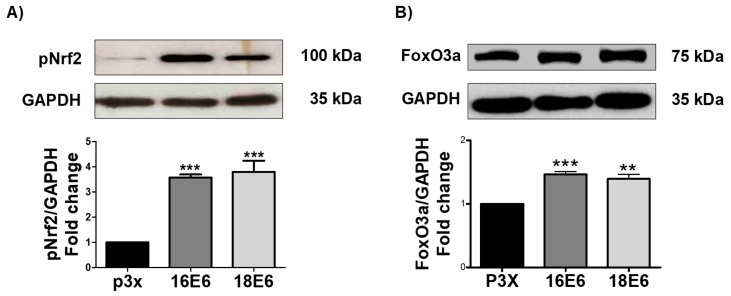
16E6 and 18E6 oncoproteins increase pNrf2 and FoxO3a levels in FaDu cells. Representative immunoblot and quantitative densitometric analysis of (**A**) nuclear factor (erythroid-derived 2)-like 2 (Nrf2) phosphorylated in serine 40 (pNrf2); and, (**B**) forkhead box O3 (FoxO3a) in FaDu E6-transfected cells. GAPDH was used as a loading control. Data are expressed as the mean ± SD. Tukey’s test ** *p* < 0.005, and *** *p* < 0.0005 vs. p3X control, n = 3.

OS response activates different transcription factors (TF) that are associated to antioxidant response, such as Nrf2 and FoxO3a. OS induces Nrf2 activation through its phosphorylation in Ser40 (pNrf2), leading Nrf2 to the nucleus [49]. Therefore, the effect of E6 oncoproteins on pNrf2 was evaluated. As shown in Figure 8A, pNrf2 (~100 kDa) augmented in the presence of 16E6 and 18E6 oncoproteins in 3.6-fold and 3.8-fold, respectively. Since glutamate-cysteine ligase modifier subunit (GCLM) is a downstream target of pNrf2, we also measured its levels. As expected, a significant increase of GCLM was found in the presence of 16E6 (2.5-fold) and 18E6 (2.9-fold), in comparison with the control cells (Figure S2A,B). Therefore, the Nrf2 pathway is active in the presence of E6 oncoproteins.

The antioxidant *N*-acetyl-l-cysteine (NAC) was added in order to demonstrate the implication of E6-induced OS in the increased pNrf2 levels. Figure S3A shows that NAC scavenge ROS induced by 16E6. Similar results were observed in cells harboring 18E6 (data not shown). Moreover, as is shown in Figure S3B, NAC treatment prevented E6 from increasing pNrf2. We conclude that ROS induced by the E6 oncoproteins are implicated in the activation of Nrf2 response.

Since Forkhead box O3 (FoxO3a) is downregulated via Akt and it is activated in response to OS, we analyzed if this TF is also affected by the E6 oncoproteins. As shown in Figure 8B, FoXO3a protein levels increased 1.5-fold in cells with 16E6 and in 1.4-fold in cells with 18E6. Therefore, we conclude that FoxO3a is active in response to OS in E6 expressing cells, being associated to the decrease in pAkt.

### 3.9. E6 Oncoproteins Increase SOD1 and SOD2 Levels and Activity

**Figure 9:**
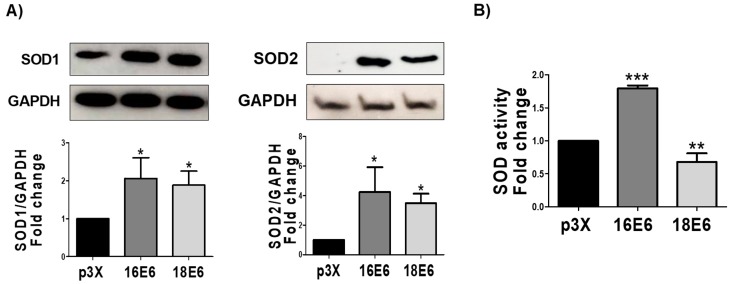
16E6 and 18E6 oncoproteins increase superoxide dismutase 1 and 2 (SOD1/2) levels in FaDu cells. (**A**) Representative immunoblot and quantitative densitometric analysis of superoxide dismutase 1 and 2 (SOD1 and SOD2); and (**B**) total SOD activity, which is augmented in FaDu 16E6 expressing cells. Glyceraldehyde 3-phosphate dehydrogenase (GAPDH) was used as a loading control. Data are expressed as the mean ± SD. Tukey’s test * *p* < 0.05, ** *p* < 0.005, and *** *p* < 0.0005 vs. p3X control, n = 3.

We measured, by immunoblot, the enzymes superoxide dismutase 1 and 2 (SOD1 and SOD2) and their activity, by spectrometry, in order to determine if an antioxidant response is activated by the transcription factors pNrf2 and FoxO3a. We observed that the 16E6 and 18E6 oncoproteins increased the protein levels of SOD1 in 2-fold and 1.9-fold, respectively; while the SOD2 levels were increased in 4.3-fold and 2.5-fold, respectively (Figure 9A). However, we found differences in SOD activation, where 16E6 promoted SOD activity, while it declined in cells with 18E6 (Figure 9B). From these results, we conclude that both E6 oncoproteins increase SOD levels, although only 16E6 augmented SOD activity.

### 3.10. 16E6 Increases Catalase Activity without Changing Catalase Levels

**Figure 10:**
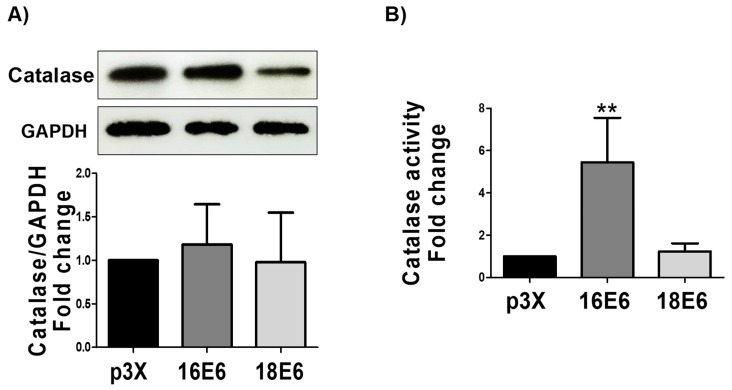
Effect of E6 oncoproteins from HPV16 and HPV18 on catalase levels and activity. (**A**) Representative immunoblot and quantitative densitometric analysis of catalase; (**B**) catalase activity in FaDu transfected cells. GAPDH was used as a loading control. Data are expressed as the mean ± SD. Tukey’s test ** *p* < 0.005 vs. p3X control, n = 3.

SOD enzymes dismute O_2_^.−^ molecules to H_2_O_2_, allowing for catalase and other peroxidases to degrade H_2_O_2_ to water. Therefore, we measured the catalase levels by immunoblot and its activity, by spectrophotometry. We found that even that E6 oncoproteins did not raise catalase expression (Figure 10A), 16E6 increased catalase activity by 5.4-fold (Figure 10B), while 18E6 had no effect. This result correlates with the activation of SOD exclusively by 16E6 oncoprotein.

### 3.11. 16E6 and 18E6 Oncoproteins Induce DNA Damage

**Figure 11:**
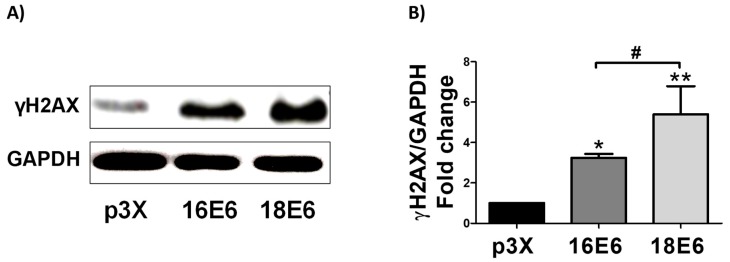
E6 oncoproteins from HPV16 and HPV18 induce DNA damage in FaDu cells. (**A**) Representative immunoblot; and (**B**) quantitative densitometric analysis of gamma H2A histone family member X (γH2AX) in FaDu-transfected cells. GAPDH was used as a loading control. Data are expressed as the mean ± SD. Tukey’s test * *p* < 0.05 and ** *p* <0.005 vs. p3X control; # *p* < 0.05 16E6 vs. 18E6, n = 3.

The DNA marker gamma H2AX (γH2AX) was analyzed in the presence of E6 oncoproteins since OS promotes DNA damage. As shown in Figure 11A,B, 16E6 and 18E6 increased DNA damage in 3.2- and 5.4-fold, respectively, in relation to the control. The induction of DNA damage was higher in 18E6 transfected cells. The cells were treated with the antioxidant NAC in order to determine whether the OS induced by E6 oncoproteins is responsible of the observed DNA damage. As shown in Figure S4A,B, NAC alleviated the DNA damage induced by E6 oncoproteins. From these results, we conclude that 16E6 and 18E6 induce DNA damage by OS induction. The highest effect observed in 18E6 expressing cells may be related to its demonstrated lowest antioxidant activity in relation to those harboring 16E6 (Figure 9B and Figure 10B).

## 4. Discussion

Mitochondria perform bioenergetic and biosynthetic processes, such as fatty acid oxidation (FAO), Krebs cycle, ETS, and cell death induction via apoptosis, as well as the synthesis of amino acids, lipids, and nucleotides [13,14]. The Krebs cycle produces NADH and flavin adenine dinucleotide (FADH_2_) that enter the ETS to generate a proton gradient in the inner membrane of the mitochondria, which is utilized to produce ATP through ATP synthase [50]. However, during this process, ROS can be generated as a side product of ETS, especially when the mitochondria is decoupled [51]. When ROS are excessively accumulated and the cellular antioxidant system is overcome, OS is produced, which leads to cell death [29]. Nevertheless, ROS in lower concentration act as secondary messengers, activating several signaling pathways and enzymes, such as Mitogen-Activated Protein Kinases, which control cell proliferation and transformation [52]

Mitochondria coordinate a wide range of functions that are critical for metabolism, growth, and cell survival, having a principal role in cancer development [15], where mitochondrial ROS and its antioxidant systems are involve in modulating cell fate. It has been shown that HNSCC HPV− and HNSCC HPV+ differ in genomic and metabolic profiles [4,6,7]. Regarding metabolic requirements, it has been reported that HNSCC HPV− prefer glucose metabolism, while HNSCC HPV+ favor mitochondrial metabolism [9,10,11]. For instance, it has been shown that HNSCC HPV− cells express genes that are related to glycolytic and OXPHOS pathways, which suggests that these cells use both mechanisms to obtain energy; while HNSCC HPV+ cells favors the expression of genes that are related to the OXPHOS pathway [53]. However, the mechanisms that are associated to mitochondrial metabolism in HNSCC HPV+ are unknown. In this work, we explored the role of HPV E6 oncoprotein in mitochondrial metabolism. We found that E6 oncoproteins from HPV16 and HPV18 raise the mitochondrial oxygen consumption, increasing the levels of mitochondrial proteins, which is associated to a reduction in pAkt (Figure 1). In line with this, De Rosa et al. showed that, in lung epithelial cells, the inhibition of pAkt activates mitochondrial metabolism and increases ETS complexes [38]. Furthermore, pAkt is downregulated in HNSCC HPV+ tumors and derived cell lines [54,55]. Moreover, it has been demonstrated the the transfection of E6/E7 in HNSCC HPV− cells decay pAkt [54].

In this study we found that E6 oncoproteins enhance cellular respiration, matching with the increase in mitochondrial protein content (Figure 2 and Figure 3). Nevertheless, although mitochondrial respiration increased, ATP-linked respiration did not show significant changes. It is possible that 16E6 and 18E6 favor the biosynthetic pathways that are implicated in the production of amino acids, nucleic acids, and fatty acids, among other biomolecules, instead of energy in ATP form during the mitochondrial metabolism [13,14,56]. We found that the increase in mitochondrial respiration was associated to the decoupling of mitochondria and the electron leak in the presence of E6 oncoproteins, which was reflected by the decline of the RCI parameter (Figure 4). In agreement with our results, Evans et al. (2016) reported that E6* boosts mitochondrial dysfunction in cervical cancer cells lines, which increases several ETS complex subunits [57]. Moreover, it has been shown that E6 deregulates different metabolic pathways [58]. Furthermore, it has been reported that mitochondrial decoupling promotes ROS production, since it favors the electron leak that partially reduce oxygen, in order to produce O_2_^.−^ [25], which contributes to OS and cell damage [59,60]. Concordantly, we found that E6 oncoproteins promote a leak respiration that is associated to the induction of ROS in mitochondria and the whole cells, triggering the observed oxidative stress (Figure 4, Figure 5, Figure 6 and Figure 7).

We also showed that the increase in ROS by E6 generated OS, as shown by the increase in carbonyl proteins and GSSG, contributing to the fall in GSH/GSSG ratio (Figure 7). We also measured the effect of E6 on these proteins since pNrf2 and FoxO3a are activated under OS conditions [26,27]. We found that the FoxO3a and pNrf2 levels increased in the presence of E6 from HPV16 and HPV18 (Figure 8), which suggests its translocation to the nucleus to regulate the antioxidant response. This was demonstrated by the increase of GCLM (Figure S2), one canonical pNrf2 target gene.

By the other side, it has been reported that pAkt inactivates FoxO3a via its phosphorylation in threonine 32 and serine 253 and 315, which is related to its activity inhibition and proteasomal degradation [61]. However, in the presence of low levels of pAkt [62], FoxO3a resides in the nucleus and triggers target genes, such as SOD2 and catalase [27]. It is probable that the activation of FoxO3a is due to low levels of pAkt and/or activation of oxidative stress by the E6 oncoproteins. Moreover, FoxO3a is an essential molecule for inducing mitochondrial biogenesis [63], which could be related to the increase in mitochondrial mass in the presence of the E6 oncoproteins.

We measured the expression of SOD1, SOD2, and catalase as the target genes of pNrf2 and FoxO3a [28,64], finding that E6 proteins was associated to the elevation of SOD2 and SOD1 levels (Figure 9). We did not find changes in catalase levels (Figure 10), which was perhaps due to epigenetic modulation of catalase by other factors, such as miRNA 30b [65], which is modulated by the E6 oncoprotein [66].

We set out to analyze this activity in the presence of E6 since the levels of antioxidant enzymes do not necessarily reflect their antioxidant activity. We found that the activity of SOD and catalase only increased in the presence of 16E6 (Figure 9 and Figure 10) and not with 18E6. This could be partially explained by differential interactions between E6 proteins from HPV16 and 18 with cellular proteins [67], which could affect the post-translational modifications of antioxidant enzymes, such as acetylation, phosphorylation, nitration, glycation, and glutathionylation [68], which are known to impact in the enzymatic activity.

It has been shown that E6 enhances OS and DNA damage in cervical cancer [21], so we evaluated whether E6 also produces DNA damage in an HNSCC model, measuring the levels of γH2AX. We demonstrated that E6 also causes DNA damage in HNSCC (Figure 11). Previous reports have shown that 16E6 upregulate proteins related to DNA damage response (DDR), such as Checkpoint kinase 1 and 2 (Chk1 and Chk2) inducing their activation [68]. The cellular hormesis, related to the increase of ROS and the response of Nrf2 and FoxO proteins, occurs continuously in cancer cells, mainly induced by the high metabolic requirements that are associated with the high rate of cell proliferation, which is the way in which hormesis determines the adaptation to environmental stress and cell survival [69].

**Figure 12:**
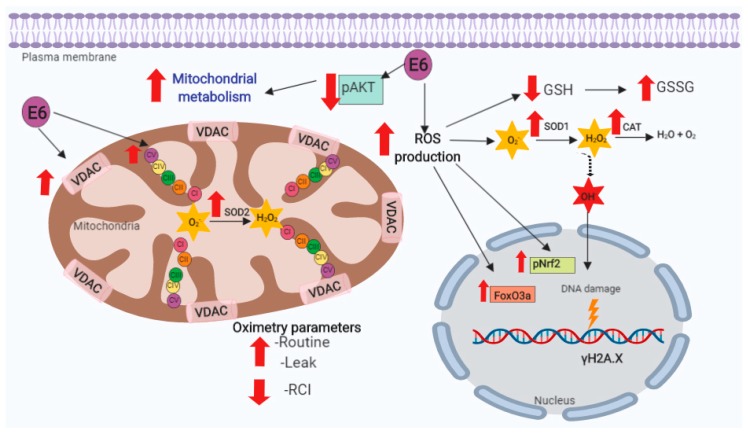
Integrative scheme. E6 oncoproteins from human papillomavirus type 16 (HPV16) and 18 (HPV18) decrease the levels of serine 473 phosphorylated-protein kinase B (pAkt), favoring the mitochondrial oxygen consumption and mitochondrial mass increase. E6 increases basal respiration (Routine), respiration leak and mitochondrial decoupling, while respiratory control index (RCI) significantly decreases, which could be associated to the increase in reactive oxygen species (ROS), oxidative stress (OS), and deoxyribonucleic acid (DNA) damage. Superoxide dismutase (SOD), catalase (CAT), glutathione (GSH), glutathione disulfide (GSSG), voltage-dependent anion channels (VDAC), superoxide anion (O_2_^.−^), hydrogen peroxide (H_2_O_2_), hydroxyl radical (OH^.−^), nuclear factor (erythroid-derived 2)-like 2 (Nrf2) phosphorylated in serine 40 (pNrf2), forkhead box O3 (FoxO3a), gamma H2A histone family member X (γH2AX), and mitochondrial complexes I to V (CI, CII, CIII, CIV, CV).The yellow ray indicates damage to DNA.

As shown in Figure 12, the E6 oncoproteins promote mitochondrial metabolism and cellular respiration; however, there is also a decoupling of mitochondria associated with increased levels of ROS presented in FaDu cells. Although ROS production is counteracted with the increase of antioxidants, these are overcome, inducing OS and DNA damage, without affecting cell viability.

Our results suggest that the induction of the mitochondrial metabolism by E6 may confer growth advantage to HNSCC HPV+ tumoral cells. However, it is necessary to understand the possible effects in mitochondrial metabolism that are given by other HPV proteins, such as E7, E2, and E1, which deserves further studies. For example, it has been shown that E2 associates with ETS complexes, inducing ROS in immortalized keratinocytes [70], while E7 decrease the ROS levels [21].

On the other hand, our data could partially explain why HPV+ tumor cells are more radiosensitive, due to the increase in ROS levels that may favor cell death in the presence of ionizing radiation. Therefore, in order to design the improved therapeutic strategies, it is important to continue investigating the role of E6, as well as other HPV proteins, in the cellular redox state to determine the differences between HNSCC HPV+ and HPV− tumors in response to treatment.

## 5. Conclusions

E6 oncoproteins from HPV16 and HPV18 promoted the rise in mitochondrial protein levels and cell respiration, which was associated with the reduction of pAkt levels. In addition, E6 generated the mitochondrial decoupling, as shown by the increase in respiration leak and by the RCI decrease, raising ROS, OS, and DNA damage (Figure 12). These results suggest that the E6 oncoproteins from HPV16 and HPV18 are inductors of mitochondrial metabolism, oxidative stress, and DNA damage in FaDu cells, which may be linked with the reported differences in biological behavior between HNSCC HPV− and HPV+ tumors.

## Supplementary Materials

The following are available online at https://www.mdpi.com/2218-273X/9/8/351/s1.
