# Supplementary material for: E6 Oncoproteins from High-Risk Human Papillomavirus Induce Mitochondrial Metabolism in a Head and Neck Squamous Cell Carcinoma Model
Source: Biomolecules. 2019 Aug 8;9(8):351. doi: 10.3390/biom9080351 (PMC6722992; doi:10.3390/biom9080351)
Supplement: Supplementary file 1 [file biomolecules-09-00351-s001.pdf]

Supplementary figures

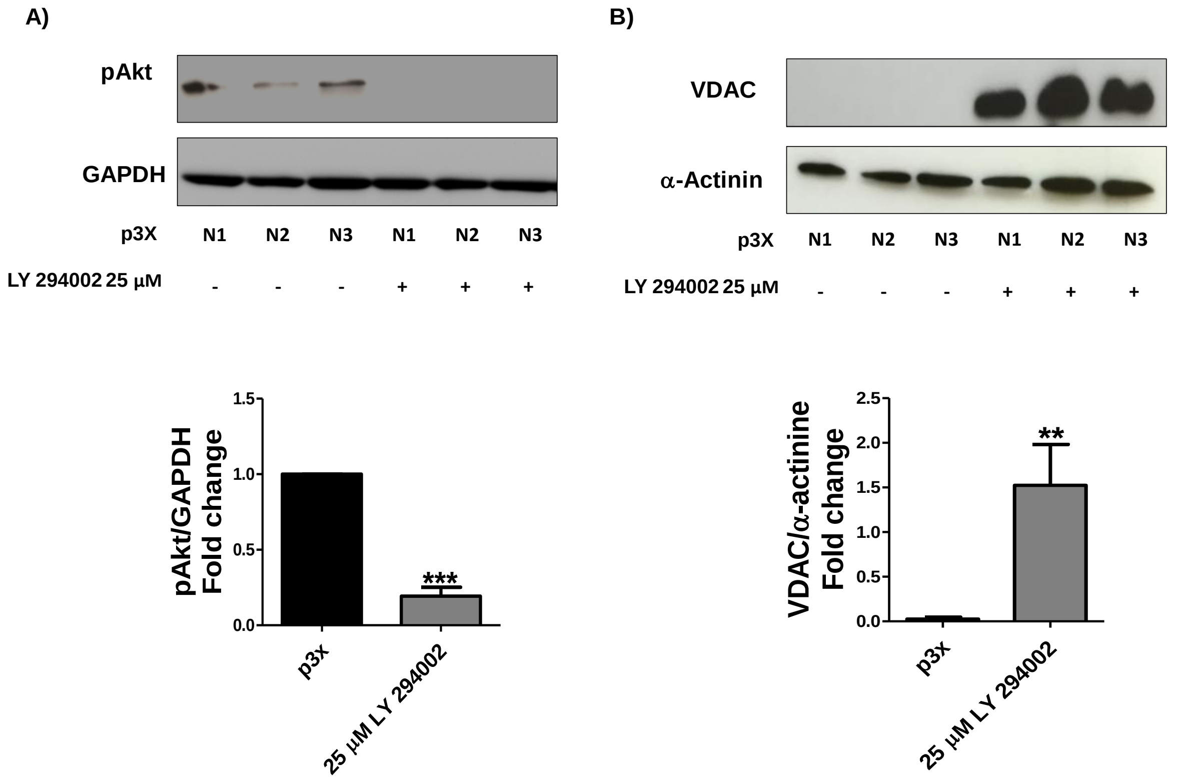

Figure S1. The pAkt inhibitor LY 294002, decreased pAkt levels and increased VDAC protein in FaDu cells transfected with p3X. At 25 µM of LY 294002, pAkt levels decreased (A), while VDAC protein levels increased in FaDu cells transfected with p3X. Data are expressed as the mean ±S D. t-student test, n=3 (N1, N2 and N3).

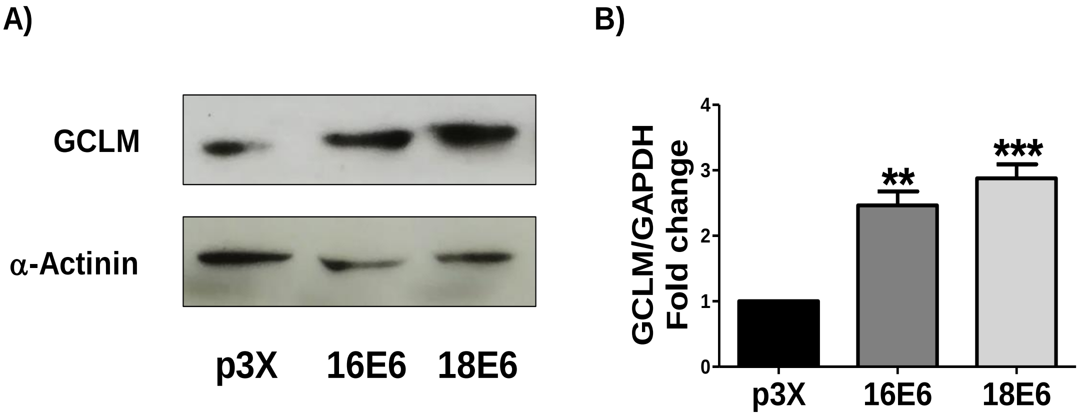

Figure S2. Protein levels of glutamate-cysteine ligase modifier subunit (GCLM) increase in FaDu cells expressing 16E6 and 18E6. A) Representative immunoblot and **B)** quantitative densitometry showing GCLM. α-Actinin was used as a loading control. Data are expressed as the mean ± SD. Tukey's test **p<0.005 and ***p<0.0005 vs p3X control, n=3.

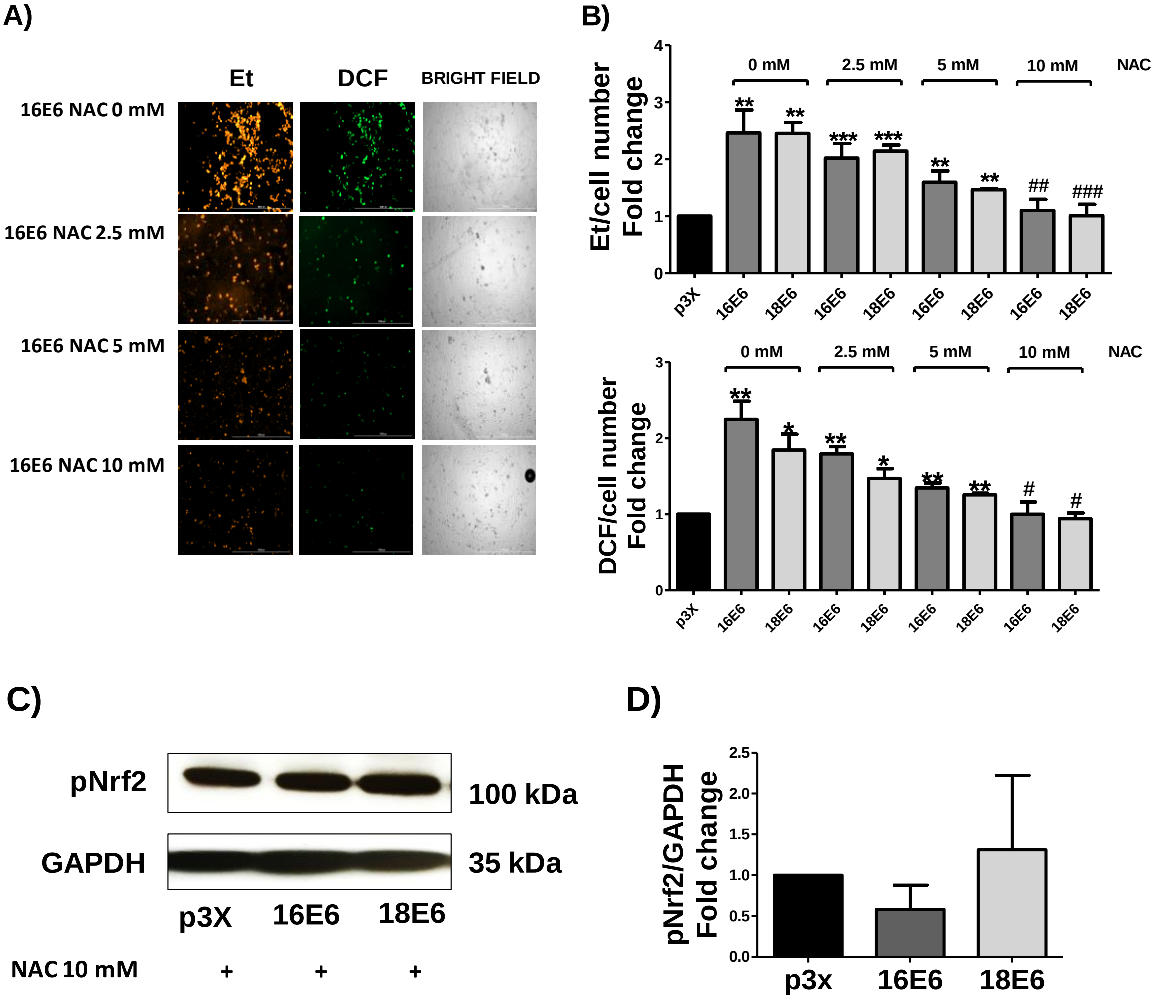

Figure S3. N-acetyl-L-cysteine (NAC) decreases ROS and prevents pNrf2 activation in FaDu cells transfected with E6 from HPV16 and HPV18. A) Representative images and **B)** quantitative data of ROS generated by FaDu cells expressing 16E6 or 18E6 oncoproteins treated with 0, 2.5, 5 and 10 mM of NAC. 10 mM of NAC significantly scavenged ROS levels in FaDu cells expressing 16E6 and 18E6 in 2.2 and 2.4-fold, respectively (measured by ethidium, Et); and 2.4 and 1.8-fold, respectively (measured by dichlorofluorescein, DCF), in comparison with no NAC added (0 mM). Quantitative data were obtained from cells expressing E6 from each viral type, comparing with p3X-transfected control cells. The mean intensity of Et and DCF fluorescence was measured using Gen5™ 3.0 software for image acquisition and quantification. The fluorescence intensity is expressed as the mean ± SD. **C)** Representative images and **D)** quantitative densitometric analysis of pNrf2 in FaDu E6-transfected cells, treated with 10 mM NAC. GAPDH was used as a loading control. 10 mM NAC prevents the increase in pNrf2 by 16E6 or 18E6. Data are expressed as the mean ± SD. Tukey's test *$p<0.05$, **$p<0.005$ and ***$p<0.0005$ vs control (p3X); t-student test, #$p<0.05$, ##$p<0.005$ and ###$p<0.0005$ 16E6 vs 18E6, n=3.

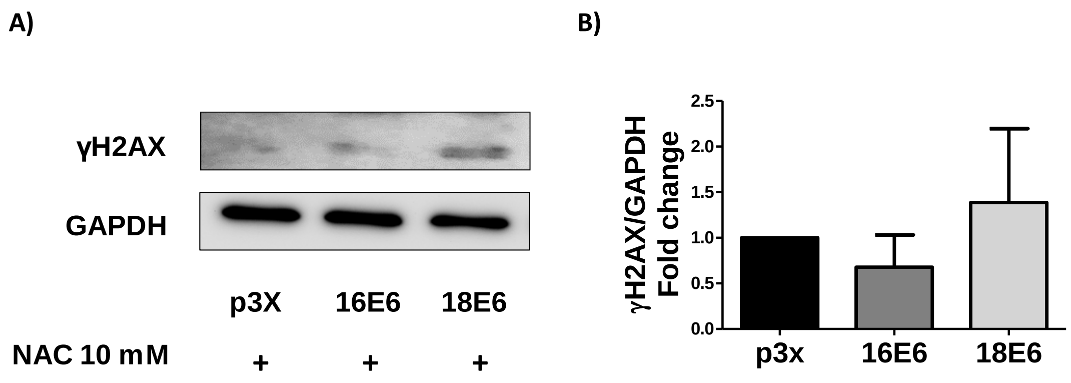

Figure S4. DNA damage induced by E6 oncoproteins in FaDu cells is alleviated by N-acetyl-L-cysteine (NAC) treatment. In the presence of NAC 10 mM E6 oncoproteins showed no effect on the levels of γH2AX, a proficient marker of DNA damage. **A)** Representative immunoblot; and **B)** quantitative densitometry, showing γH2AX. Glyceraldehyde 3-phosphate dehydrogenase (GAPDH) was used as a loading control. Data are expressed as the mean ±SD. Tukey's test, n=3.
